# Neratinib for Extended Anti-HER2 Therapy in Early Breast Cancer

**Published:** 2018-04-01

**Authors:** G. Thomas Budd, Wendy H. Vogel

**Affiliations:** 1 Taussig Cancer Center at Cleveland Clinic, Cleveland, Ohio;; 2 Wellmont Cancer Institute, Kingsport, Tennessee

## Abstract

Neratinib following the completion of trastuzumab has been shown to reduce the risk of disease recurrence for patients with HER2-positive early breast cancer in whom extended adjuvant therapy seems advisable. In clinical trials, the most common side effect of neratinib and other anti-HER2 therapies was diarrhea. Presenters at JADPRO Live clarified the findings of trials studying the benefit of differing prophylactic regimens with loperamide, budesonide, and colestipol.

Neratinib (Nerlynx) is now available for the treatment of human epidermal growth factor receptor 2 (HER2)–positive early breast cancer in patients in whom extended adjuvant therapy seems advisable. At 2017 JADPRO Live, the use of this agent and the management of side effects were discussed by G. Thomas Budd, MD, of Taussig Cancer Center at the Cleveland Clinic in Ohio, and Wendy H. Vogel, MSN, FNP, AOCNP®, of Wellmont Cancer Institute in Kingsport, Tennessee.

"Neratinib is the new kid on the block. It’s different than most other anti-HER2 agents," Dr. Budd said. It is an oral tyrosine kinase inhibitor that, in addition to targeting HER2, also targets HER1 and HER4. It binds irreversibly at the adenosine triphosphate (ATP) binding site. Its main toxicity is diarrhea, but this can be controlled with proper management.

The rationale for extended adjuvant therapy with neratinib—following a year of trastuzumab (Herceptin)—is the fact that 25% of women treated with adjuvant trastuzumab have recurrences after 8 to 10 years, and this risk is not reduced by longer durations of adjuvant trastuzumab treatment. To the contrary, 1 year of neratinib following completion of trastuzumab has been shown to reduce the risk of disease recurrence, he said.

## ExteNET TRIAL

The phase III ExteNET trial was a multicenter, randomized, double-blind, placebo-controlled study in 2,840 patients who received neratinib (240 mg/day) or placebo after completing chemotherapy and 1 year of trastuzumab (Martin et al., 2017). Patients were enrolled up to 2 years after trastuzumab therapy. (Ideally, women should start the drug within a year of completing trastuzumab, but they are candidates for the drug up to 2 years later.)

"Patients overall did well, but those who also took neratinib did a little better," Dr. Budd said (see Table 1). "Neratinib reduced the risk of recurrence by about one-fourth." Invasive disease-free survival was 90.2% in the neratinib arm vs. 87.7% in the placebo arm (hazard ratio [HR], 0.73; *p* = .008). Recurrences in the central nervous system (CNS) were numerically less (1.3% vs. 1.8%), although the difference was not statistically significant (*p* = .333). "Neratinib seems to have some activity against CNS metastases, but they were not common in either arm of this trial," he commented.

**Table 1:**
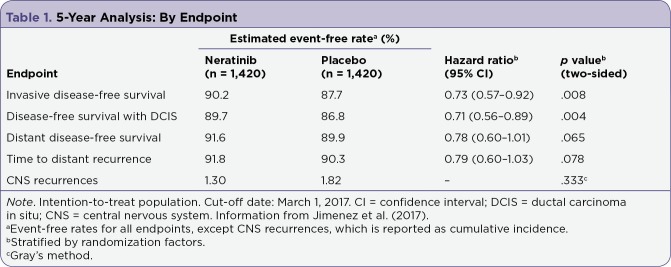
5-Year Analysis: By Endpoint

The drug demonstrated a sizable advantage in patients with hormone receptor–positive tumors (HR, 0.51), a finding that the investigators are still trying to explain. "Hormone receptor–positive patients seem to derive particular benefit," Dr. Budd commented.

The principal side effect in the study was diarrhea, which was grade 1 to 2 in 55% and grade 3 in 40% of neratinib-treated patients. "At the time this trial was done we were not that familiar with the management of this toxicity, and in our phase I study diarrhea was a real problem. There was a steep learning curve," he said. "It’s worse at the beginning of treatment, and it gets better. You have to learn to control it, to get the patient through this period, so she can enjoy the benefit of this therapy."

## ESTABLISHED PRINCIPLES AND QUESTIONS BEING STUDIED IN EARLY-STAGE HER2-POSITIVE DISEASE

Older studies have established several key principles in treating early-stage HER2-positive breast cancer:

- Adding trastuzumab to paclitaxel after doxorubicin and cyclophosphamide yields a sustained reduction in cancer recurrence; recurrence risk continues at least to year 20 after adjuvant therapy is finished.
- Neoadjuvant pertuzumab (Perjeta), given with trastuzumab and docetaxel, improved pathologic complete response rates in the NeoSphere trial. American Society of Clinical Oncology guidelines recommend trastuzumab/pertuzumab/taxane for first-line neoadjuvant treatment.
  Two of the most important clinical trials are evaluating new regimens in the adjuvant setting:
- KATHERINE: Trastuzumab emtansine (Kadcyla) vs. trastuzumab as adjuvant therapy in patients with residual tumor in the breast or nodes after neoadjuvant therapy. Patients are randomized to trastuzumab emtansine at 3.6 mg/kg or trastuzumab at 6 mg/kg IV every 3 weeks for 14 cycles.
- KAITLIN: Following anthracycline-based adjuvant therapy, trastuzumab emtansine plus pertuzumab vs. trastuzumab plus pertuzumab and a taxane. Dosing is tratuzumab emtansine at 3.6 mg/kg and pertuzumab at 420 mg IV every 3 weeks, and trastuzumab at 6 mg/kg and pertuzumab at 420 mg IV every 3 weeks in combination with a taxane.

## DIARRHEA ASSOCIATED WITH ANTI-HER2 THERAPY

Ms. Vogel discussed the management of diarrhea, which is the most common side effect of anti-HER2 therapy and occurs to some degree in the vast majority of patients on pertuzumab, lapatinib (Tykerb), and neratinib. Severe cases can be life-threatening. In the least, the diarrhea can lead to dose reductions and delays, reduced treatment adherence, diminished quality of life, and increased cost of care.

There are several predictive factors for diarrhea grade 2 and higher: advancing age, occurrence of grade 1 diarrhea in a prior cycle (2-fold increased risk), and, for reasons that are not clear, treatment that is initiated in the springtime (2-fold increased risk; Dranitsaris & Lacouture, 2014).

## MANAGEMENT OF DIARRHEA IN THE EXTENDED ADJUVANT SETTING

The CONTROL trial established the benefit of loperamide prophylaxis in preventing diarrhea during neratinib treatment (Ibrahim et al., 2017). It was a phase II trial in which 135 women with early HER2-positive breast cancer were treated with neratinib at 240 mg/day (and endocrine therapy as indicated). All patients completed trastuzumab-based therapy ≤ 1 year prior to enrollment. The study evaluated a scheduled regimen of loperamide prophylaxis, given for two cycles (then as needed from day 57 onward). The outcomes were compared to toxicity observed in the ExteNET trial when loperamide was given as needed.

In the original protocol, the following stepped-down regimen was followed: loperamide at 16 mg/day (4 mg plus 2 mg every 4 hours on day 1); loperamide at 12 mg/day (2 mg every 4 hours on days 2–3); loperamide at 6–8 mg/day (2 mg every 6 or 8 hours on days 4–56); and finally, starting on day 57, loperamide as needed.

The dosing schedule was modified as follows: loperamide at 16 mg/day (4 mg plus 4 mg given three times a day on day 1); 12 mg/day (4 mg three times a day on days 2–14); loperamide at 8 mg/day (4 mg twice a day) on days 15 to 56; and as needed from day 57 onward.

Subsequently, in 2017, two additional cohorts were added, based on preclinical studies. These arms evaluated loperamide plus budesonide (9 mg/day, extended release tablets) and colestipol during the first cycle. Budesonide is a locally acting corticosteroid believed to target the inflammation associated with neratinib-induced diarrhea. Colestipol is a sequestrant believed to target the bile acid malabsorption that occurs with neratinib-induced diarrhea.

## EFFECTIVE PROPHYLAXIS IN CONTROL TRIAL

The study showed that loperamide prophylaxis is very effective in preventing serious diarrhea. Most treatment-emergent diarrhea occurred during the first cycle; in fact, 75% of all diarrheal events occurred in the first 4 weeks and more than half of all grade 3 events occurred within the first week. There were no grade 3 events after the first cycle and no grade 4 events at all, Ms. Vogel reported.

The modified schedule was more effective than the original protocol in preventing diarrhea, and the addition of budesonide was further preventative (Table 2). All the CONTROL arms had substantially less diarrhea than was observed in the ExteNET trial in which 95% of patients, on as-needed dosing, had diarrhea, and 39.8% had grade 3 diarrhea.

**Table 2:**
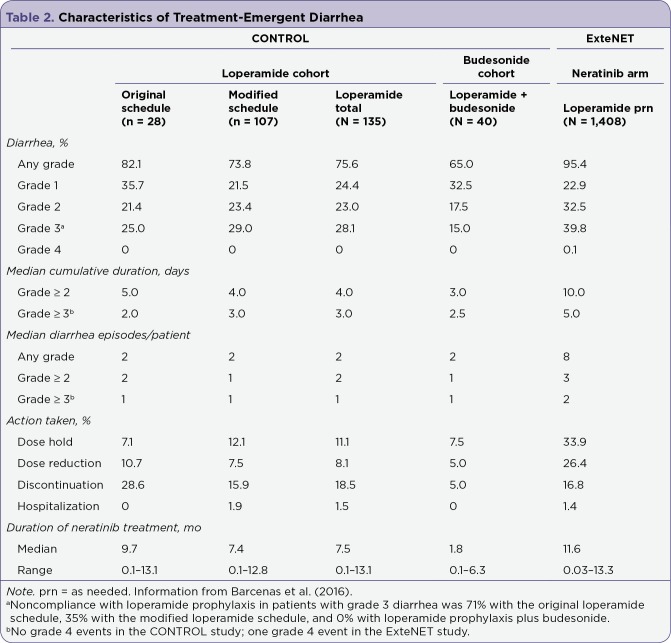
Characteristics of Treatment–Emergent Diarrhea

Preliminary data suggest that adding budesonide to loperamide prophylaxis may further diminish the duration and number of episodes of diarrhea, as well as decrease the number of neratinib dose holds, reductions, and discontinuations. The results achieved with the addition of colestipol will be reported later.

"ExteNET demonstrated diarrhea that was often high-grade, highest in month 1 and persistent in a large proportion of patients in months 2 to 12. In the CONTROL study cohorts, diarrhea was characterized by a lower percentage of high-grade events in month 1 and a much lower incidence in months 2 to 12," Ms. Vogel said. "We saw a nice decrease in diarrhea when we gave loperamide on a scheduled basis, in higher doses, and with the addition of the corticosteroid."

She added that patients do adapt to neratinib, and the high grades of diarrhea are seen early and do not typically recur. It is important to act promptly to prevent and control diarrhea to maximize the drug’s benefit to patients, and it is imperative to educate patients on the optimal dosing of prophylactic antidiarrheals.

## ROLE OF THE ADVANCED PRACTITIONER IN MANAGING PATIENTS ON ANTI-HER2 THERAPY

Ms. Vogel also emphasized the role of advanced practitioners in managing patients on anti-HER2 therapy. They help in choosing the appropriate drug for the right patient, in assimilating the data to explain these choices to patients, in educating patients about treatments, and in baseline assessments.

"Patient education is key—not just about the disease process and treatment, but about self-management of potential toxicities," she said. "Patients must be proactive, not reactive. They need to know when and to whom to report toxicities."

Advanced practitioners should also oversee triage calls and know how to grade toxicity. "Diarrhea from HER2 therapy differs from chemotherapy-induced diarrhea," she said. "It’s not just ’take two loperamide and call me in the morning.’ You have to be more alert."

She advised having a high index of suspicion for problems and to make face-to-face assessments. Physical examination should include assessment of the abdomen and rectal areas, weight, vital signs, nutrition (with referrals to dieticians as needed), and electrolytes.

"Be alert to unique toxicities. Patients may not connect a side effect with treatment, because they believe that oral medicines are safer. Be aware that adherence to oral cancer drugs can also be an issue," she added.
